# Implementing nurse-initiated and managed antiretroviral treatment (NIMART) in South Africa: a qualitative process evaluation of the STRETCH trial

**DOI:** 10.1186/1748-5908-7-66

**Published:** 2012-07-16

**Authors:** Daniella Georgeu, Christopher J Colvin, Simon Lewin, Lara Fairall, Max O Bachmann, Kerry Uebel, Merrick Zwarenstein, Beverly Draper, Eric D Bateman

**Affiliations:** 1 Knowledge Translation Unit, University of Cape Town Lung Institute, University of Cape Town, George Street, Mowbray, 7700, Cape Town, South Africa; 2 Centre for Infectious Disease Epidemiology and Research (CIDER), School of Public Health and Family Medicine, Falmouth Building, Faculty of Health Sciences, University of Cape Town, Observatory, 7925, Cape Town, South Africa; 3 Norwegian Knowledge Centre for the Health Services, Pilestredet Park 7, 0176, Oslo, Norway; 4 Health Systems Research Unit, Medical Research Council, Francie van Zijl Drive, Tygerberg, 7505, South Africa; 5 Department of Medicine, Faculty of Health Sciences, University of Cape Town, Old Main Building, Groote Schuur Hospital, Observatory, 7925, Cape Town, South Africa; 6 Norwich Medical School, University of East Anglia, Earlhan Road, Norwich, NR4 7TJ, United Kingdom; 7 Department of Internal Medicine, Faculty of Health Sciences, University of the Free State, Nelson Mandela Drive, Bloemfontein, 9301, South Africa; 8 Sunnybrook Research Institute and Department of Health Policy, Management and Evaluation, Health Sciences Building, University of Toronto, College Street, Toronto, M5T 3M6, Canada

**Keywords:** Antiretroviral treatment, NIMART, South Africa, Primary healthcare, Nurse training, Process evaluation, PALSA PLUS

## Abstract

**Background:**

Task-shifting is promoted widely as a mechanism for expanding antiretroviral treatment (ART) access. However, the evidence for nurse-initiated and managed ART (NIMART) in Africa is limited, and little is known about the key barriers and enablers to implementing NIMART programmes on a large scale. The STRETCH (Streamlining Tasks and Roles to Expand Treatment and Care for HIV) programme was a complex educational and organisational intervention implemented in the Free State Province of South Africa to enable nurses providing primary HIV/AIDS care to expand their roles and include aspects of care and treatment usually provided by physicians. STRETCH used a phased implementation approach and ART treatment guidelines tailored specifically to nurses. The effects of STRETCH on pre-ART mortality, ART provision, and the quality of HIV/ART care were evaluated through a randomised controlled trial. This study was conducted alongside the trial to develop a contextualised understanding of factors affecting the implementation of the programme.

**Methods:**

This study was a qualitative process evaluation using in-depth interviews and focus group discussions with patients, health workers, health managers, and other key informants as well as observation in clinics. Research questions focused on perceptions of STRETCH, changes in health provider roles, attitudes and patient relationships, and impact of the implementation context on trial outcomes. Data were analysed collaboratively by the research team using thematic analysis.

**Results:**

NIMART appears to be highly acceptable among nurses, patients, and physicians. Managers and nurses expressed confidence in their ability to deliver ART successfully. This confidence developed slowly and unevenly, through a phased and well-supported approach that guided nurses through training, re-prescription, and initiation. The research also shows that NIMART changes the working and referral relationships between health staff, demands significant training and support, and faces workload and capacity constraints, and logistical and infrastructural challenges.

**Conclusions:**

Large-scale NIMART appears to be feasible and acceptable in the primary level public sector health services in South Africa. Successful implementation requires a comprehensive approach with: an incremental and well supported approach to implementation; clinical guidelines tailored to nurses; and significant health services reorganisation to accommodate the knock-on effects of shifts in practice.

**Trial registration:**

ISRCTN46836853

## Background

### Task-shifting in large-scale public ART programmes

The scale-up of public sector antiretroviral treatment (ART) programmes for HIV/AIDS in Southern Africa has created additional workload and organisational challenges, deepening concerns about the ongoing shortage of human resources for health. While most programmes in South Africa have used a model of physician-initiated and managed ART, there are insufficient physicians in the public sector, which provides the vast majority of HIV/AIDS care in South Africa, to take this approach to national scale. Physician-led ART programmes also tend to shift HIV/AIDS care from the comprehensive primary care level to selective disease-specific programmes in hospitals and larger clinics, resulting in fragmented services for patients.

‘Task-shifting’ from physicians to nurses has been proposed as one response to the challenge of delivering large-scale, sustainable, and effective ART programmes in resource-constrained contexts [1-4]. Task-shifting of roles and responsibilities for HIV care and treatment in this context can take a number of forms depending on how services are structured [5]. In the context of HIV/AIDS care the term ‘nurse-initiation and management of ART (NIMART)’ is proposed. In its fullest sense, NIMART involves nurse-initiation of patients onto ART, re-prescription for patients stable on ART, and appropriate referral to physicians as needed. Given the shortages of physicians in most low- and middle-income (LMIC) countries with large-scale ART programmes, there is an emerging consensus that some form of NIMART, or ART provision by other non-physicians [6], will be required to achieve ART coverage. There is limited evidence, however, on the feasibility and effectiveness of NIMART on a large-scale within weak health systems and much of the available evidence is of limited applicability to resource-constrained settings [1,7].

### The free state ART programme, PALSA PLUS, and the STRETCH trial

In 2004, concerns that the vertical nature of the ART programme in South Africa’s Free State province was draining resources from and fragmenting care at primary healthcare (PHC) facilities prompted the provincial Department of Health to agree to the implementation of the PALSA PLUS (based on PALSA, the Practical Approach to Lung Health in South Africa) programme. PALSA PLUS consists of a comprehensive set of user-friendly, evidence-based, algorithm-driven syndromic guidelines for the PHC nurse clinical management of respiratory diseases and HIV/AIDS [8-13], implemented through educational outreach training and support to all PHC staff at each facility.

PALSA PLUS was well-received and effective [13] in the Free State, but health managers remained concerned with the persistently high mortality among ART-eligible patients awaiting treatment and the chronic undersupply of physicians available to provide ART [14]. In 2005, the Department of Health asked for the incorporation of NIMART into its existing PALSA PLUS guidelines and training. Because of concerns regarding the ability of nurses to prescribe ART safely and the absence of a clear national policy on nurse-initiation, it was decided to evaluate this rollout of NIMART with a pragmatic randomised controlled trial and qualitative process evaluation. The subsequent STRETCH (Streamlining Tasks and Roles to Expand Treatment and Care for HIV) trial evaluated the safety and effectiveness of NIMART and examined how best to implement NIMART in a resource-constrained, publicly-funded health system [15,16].

The trial was conducted between October 2007 and June 2010 and the STRETCH intervention and its implementation are described in more detail elsewhere [17]. As in other provinces, the Free State ART programme was implemented as a vertical disease-specific programme with distinct funding, staffing, accreditation, and administration processes. In the Free State, two kinds of ART sites were created: nurse-led ‘assessment sites’ in selected PHC facilities where patients could be screened and prepared for ART and receive monthly supplies of ART, and physician-led ‘treatment sites’ located in larger facilities where patients were referred for initiation of ART and six-monthly reviews of ART prescriptions. Patients diagnosed HIV-positive at a PHC clinic offering basic adult, maternal, and child healthcare would be referred to ART nurses at an ‘assessment site’ either within that same clinic or at another clinic for blood tests, routine HIV care, and preparation for ART. Once eligible for ART, patients would be seen at a more distant ‘treatment site,’ often on hospital premises, for treatment initiation by a physician. Once initiated, patients would then receive monthly supplies of ART at the assessent site and be referred every six months back to the treatment site for treatment review by the physician. This approach continued in control clinics.

The STRETCH intervention evaluated here was a multifaceted health systems intervention that aimed to improve access to ART by moving assessment and treatment nearer to the patients’ homes by providing both services in lower-level clinics. The intervention devolved clinical responsibility for ART from physicians to nurses for selected patients (task-shifting), and decentralised and integrated other aspects of HIV and ART care (e.g. routine HIV care and pre-treatment assessment) into primary healthcare services.

All 31 ART assessment sites from all five districts of the Free State were randomised to receive the intervention (n = 16) or to continue with their current ART service provision model (n = 15). Primary outcomes for the trial were mortality for those needing but not yet receiving ART (cohort 1) and viral load suppression for those already on ART for six months or longer (cohort 2) [15]. The trial results are reported elsewhere [16].

STRETCH was implemented in three phases to facilitate a gradual increase in nurse skill and confidence as well as ensure sufficient time for the necessary logistical and management adjustments required by NIMART in the intervention clinics (Table1). Phase one involved clinic orientation, preparation, and initial decentralisation of some forms of routine HIV care. Phase two involved the consolidation of the decentralisation of routine HIV care, ART monitoring, and ART re-prescription by nurses. Phase three involved triage, referral, and in selected cases initiation of ART by STRETCH nurses.

**Table 1:** Summary of phased implementation of the STRETCH intervention

**Phase 1**	**Phase 2**	**Phase 3**
Site preparation	Decentralisation of HIV care and ART monitoring	Initiation of ART treatment by STRETCH nurses
- Implement PALSA PLUS and STRETCH guideline training with all clinic nurses using a middle- manager trainer delivering case-scenario based training	- Consolidate efforts to decentralise elements of routine HIV care such as initial laboratory workup, drug readiness training and monthly supply of ART to PHC nurses	- Triage by STRETCH nurses of all clients referred for ART and initiation of treatment in new patients without clinical complications requiring referral
- Convene support team composed of current facility staff and local management for each STRETCH facility to initiate systems changes for Phases 2 and 3	- ART monitoring decentralised to nurses at STRETCH facilities	- Referral of patients not eligible for nurse-initiation to physician
- Start decentralisation of routine HIV care e.g. VCT and CD4s to be done at local PHC clinic	- Re-prescription of ART by STRETCH nurses for previously-initiated patients stable for six months or more	- STRETCH support team meetings continue
	- Weekly STRETCH support team meetings	

Intervention sites were allowed to progress through the three phases at their own pace and tailor the intervention to their local context. Given the complexity of the intervention, STRETCH sites were provided with a ‘STRETCH Implementation Toolkit,’ a 30-page document that included a decentralisation checklist, a detailed description of the study and its phases, descriptions of the changing roles for health workers, advice on communicating the study aims and procedures to patients and communities, and the relevant contact information and institutional authorisations for the study.

Support for nurses at STRETCH sites and for the other staff and managers took several forms. STRETCH trainers offered ongoing, on-site support to nurses. Physicians working on site, at referral hospitals, or at a Centre for Excellence in HIV care at a large central referral hospital were tasked with supporting and mentoring nurses. STRETCH support teams were set up for each site to provide logistical and management support. Finally, the STRETCH trial coordinator (KU), a family practitioner with experience in tuberculosis and HIV, assisted with initial training, ongoing clinical support to sites, and ongoing management and logistical troubleshooting at all levels of the health system.

### Qualitative process evaluation of the STRETCH intervention

A qualitative process evaluation was conducted alongside the STRETCH trial. The aims of this qualitative process evaluation were to explore the experiences, attitudes, and practices of a wide variety of stakeholders during the process of programme implementation and to develop an understanding of the impact of broader structural and contextual factors on the implementation process. Study research questions centred around: patient, healthcare provider, and manager perceptions of the STRETCH programme; impacts of STRETCH on the provision of HIV/AIDS care and primary care; changes in health provider roles, attitudes, and patient relationships; impacts of the implementation context on trial outcomes; and the impacts of the intervention on an integrated health systems approach to care.

## Methods

### Study design

This study was a process evaluation using three qualitative research methods: in-depth interviews with key informants, focus group discussions, and observations. This research design permits an assessment of the fidelity of the implementation of the intervention under study, a detailed description of the processes, relationships, and contexts involved in the delivery of complex health system interventions, and the identification of ‘critical elements that have contributed to programme successes and failures.’ It thus addresses the ‘black box’ problem in interpreting trial results by improving understanding of the mechanisms that connect particular interventions to particular outcomes [18].

### Population and sampling

STRETCH was implemented in 16 randomly selected assessment sites across the five districts of the Free State Province. Each district contained intervention and control sites delivering ART along with smaller PHC clinics that referred patients to these treatment and assessment sites. For the process evaluation, we sampled three of these districts and collected data from purposively sampled intervention, control, and PHC clinics that made referrals to assessment sites within each district. We later conducted in-depth interviews at two further intervention sites in a fourth district in order to investigate emerging difficulties in that district. The fifth district was not included due to very high patient loads and site-level reluctance to participate at the time.

Purposive sampling was used to ensure that the clinics chosen for detailed study represented both urban and rural sites of different sizes with varying degrees of management and physician support and varied human resources profiles [19]. Three of the five intervention sites were in rural areas with varying levels of physician support. The Free State borders six other provinces and Lesotho, and a significant degree of migration of patient populations was reported between provinces and sites.

### Data collection

Data were collected primarily by DG, a qualitative researcher with a background in nursing. Interviews with health department managers and other key stakeholders were conducted jointly by CJC, SL, and DG in respondents’ offices or telephonically. Trainer, facility manager, nurse, and patient interviews and focus groups were conducted at the clinics. Patient focus groups were conducted in participants’ home language, seSotho, by a translator/researcher, and then transcripts of recordings were translated from seSotho into English. The remaining observation, interview and focus group data were collected in English, which was the language in which training was conducted. Table2 provides further information on data collection and participants.

**Table 2:** Data collection methods

**Data collection methods**	**Participants**	**Number/Sessions**
Focus Group Discussions (FGD)	Pre-implementation FGD with nurses	1
	STRETCH site nurses	3
	Control site nurses	3
	PHC site nurses	3
	STRETCH site patients	2
	Control site patients	2
	PHC site patients	2
In-depth and Key Informant Interviews	STRETCH nurse trainers	3
	STRETCH facility managers	5
	STRETCH site physicians	2
	STRETCH trial coordinator	6
	Local, district and provincial health managers and other key stakeholders	10
Observation	Quarterly support workshops for nurse-trainers	5
	Trial coordinator support visits to clinics	3

### Data analysis and ethics

All data were audio-recorded, transcribed verbatim, and then analysed thematically. Data were managed and coded using NVivo 8 qualitative data analysis software. DG and CJC read through the transcripts and developed an initial coding framework. They then started coding transcripts and twice, together with SL, revisited the overall coding framework and revised it. This facilitated the organizing of these codes into broader themes which were elaborated iteratively by moving back and forth between the coded data and the emerging themes. For each key analytic theme, data extracts were identified on the basis of being representative and/or interesting illustrations of an emerging issue [20]. All negative instances of the findings were discussed and accounted for.

Data analysis considered themes that both ran across all sites (irrespective of intervention/control status) as well as themes that were specific to STRETCH intervention sites. In the results sections when the term ‘all clinics/sites’ is used, this refers to all three types of primary healthcare facilities in the study—intervention, control, and PHC clinics.

Reliability and validity of the analysis was enhanced through iterative data collection, the use of a multi-method design incorporating interviews, focus groups and observations, and the ongoing discussion of findings within the research team for scrutiny and feedback [21,22].

The majority of the qualitative data analysis was completed before the quantitative results of the trial were available. Once preliminary quantitative findings were available, a further round of data analysis was conducted in order to contextualise and help interpret those findings. The findings and interpretations reported below, however, were developed without reference to these quantitative outcomes in order to avoid bias in the process of analysis. An integrated analysis of the qualitative and quantitative findings will be reported elsewhere.

Ethical review of the trial and the process evaluation was obtained from the Health Sciences Faculty Human Research Ethics Committees of the University of Cape Town (142/2007) and the University of the Free State (ETOVS 75/07), both of which approved the study. All the requirements of the Helsinki Declaration of 2008 were fulfilled. Informed consent was obtained in writing from all participants and any information that might allow individuals to be identified has been deleted to ensure their anonymity. Those participating in the study were not paid. Permission for entry into healthcare facilities was obtained from the Free State Department of Health.

## Results

The STRETCH trial showed that the expansion of primary care nurses’ roles to include ART initiation and re-prescription can be done safely, and can improve health outcomes and quality of care for the duration of care covered by the trial. Nurse-initiation and re-prescription did not, however, reduce time to ART or mortality [16]. The results reported below present the key findings emerging from the qualitative evaluation of this trial. The first three sections report on the general acceptability and fidelity of the implementation process. The last four sections highlight four key factors—pharmacy, human resources, clinical support, and local management input—that affected the implementation of STRETCH.

As noted above, some of the findings reported below were common to both intervention and control sites and others were specific to the STRETCH sites. There were no major differences observed between the STRETCH and control sites along several important dimensions, including management effectiveness, health systems and infrastructure constraints, forms of service organisation, and presence of decentralisation/integration processes. Control sites faced all of the same health systems pressures that STRETCH sites reported, they showed the same kinds of variation across clinics, and they also reported some decentralisation and integration of HIV services independent of the STRETCH intervention.

### General acceptability of the STRETCH intervention

There was generally good commitment to STRETCH among management, trainers, clinic staff, and patients. Nurses were comfortable with and enthusiastic about the opportunity to be involved more directly in providing life-saving treatment:

"‘We can ‘STRETCH’ ourselves very far. This is our sisters, our brothers, our mothers we are nursing. Otherwise we would have gone to Australia or UK to work.’ [STRETCH nurse and trainer]"

STRETCH was also seen as acceptable, feasible, and, indeed, urgently needed by staff at the control sites.

Management and political support at the provincial level were strong, though the intensity of involvement of management at clinic level was variable, reflecting a broader weakness in health management in primary care [11,23]. The attitude among physicians was reported to be more mixed: participants felt that the majority supported decentralisation and nurse initiation of ART, but a significant minority were perceived to be uncertain about the ability of nurses to manage and appropriately refer more complex cases.

Patients were very supportive of STRETCH and appreciated both the improved access to care and the reduction in travel costs and time once on treatment now that they were able to receive HIV care and ART nearer to their homes. STRETCH nurses argued that the decreases in patient travel facilitated by decentralised care were a major factor in patients’ overall acceptance of the intervention. Most patients were satisfied to have their care managed by nurses, but there was still a tension for some between wanting ART to remain a separate service and wanting the benefits of a mainstreamed ART programme:

"‘We don’t have a problem waiting with everyone but we want our files separated and our nurse should just call our names and we go to our specific room.’ [patient]"

"‘Again we want to have our own nurse. Sometimes we experience personal problems that we would like to discuss with our nurse but it is not easy if today you are seen by this one and next time is that one.’ [patient]"

Some patients requested their own section of the clinic where they could avoid the long waiting lines in the general clinic and could be seen by a nurse familiar to them. Others wanted to continue to receive their care from a physician because of both the physician’s higher clinical status as well as the fact that only physicians can medically certify social grant applications, a key source of income for people living with HIV/AIDS in South Africa.

### Training and support: strong foundations but inconsistent follow-up

Nurses responded positively to the initial training on STRETCH’s guideline-based approach. Their prior training on and experience with PALSA PLUS was important in facilitating their understanding of the STRETCH approach and its implementation. There was significant variation, however, in the quality and quantity of the ongoing support provided to intervention sites. Some staff felt that STRETCH trainers lacked direct clinical experience and perceived that they did not have sufficient time in their work schedule to travel regularly to sites to provide support. These factors prevented some trainers from fulfilling the role that staff expected of them. Support and mentorship from physicians also varied greatly. Where strong support was available, nurses were more likely to report that they had developed clinical confidence.

Active local management support for implementation also varied, and many participants reported that the trial coordinator often had to step in to address gaps in logistical and management support to sites. This ongoing support should ideally have been provided by the local STRETCH ‘support teams’ composed of local area and site managers, ART and pharmacy coordinators, and physicians and nurses. Some of these teams, however, struggled with leadership and logistical challenges (see below) and did not function effectively.

### Variations in the pace of implementing NIMART

**Figure 1:**
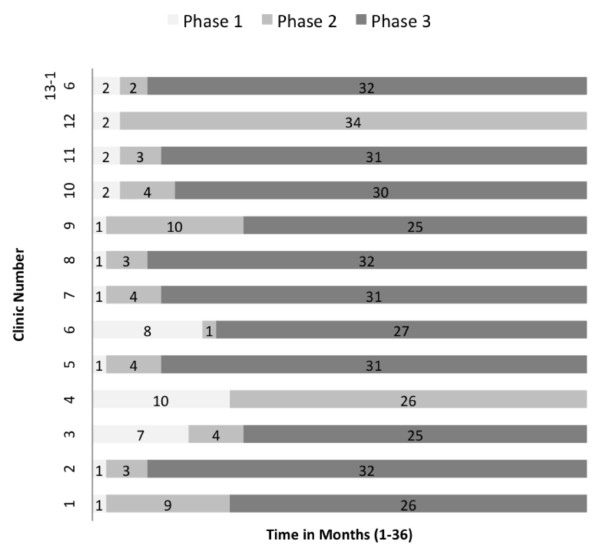
Progression of clinics through the three STRETCH implementation phases.

The pace with which sites progressed through the three phases of STRETCH—training, re-prescription and decentralisation, and nurse-initiation—varied considerably (Figure1). Though the STRETCH Toolkit recommended a four- to six-month timeline for progressing through these phases, clinics were also encouraged to adapt the pace of roll-out to suit local resources and conditions. However, a number of sites struggled to meet the basic requirements for progressing through the phases. As a result, the start dates for nurse-initiation were spread over 10 months with only six of the 16 sites starting in January 2008 as planned. Two sites never progressed from the second to the third phase (though they remained in the trial).

A number of factors were consistently reported as having influenced the pace with which intervention sites progressed. Some sites had difficulties in implementing the decentralisation aspect of STRETCH despite the fact that ad hoc decentralisation of routine HIV care services to PHC clinics had already occurred in a number of intervention and control sites prior to the STRETCH trial. Barriers to decentralising care included high staff turnover, resource and logistical constraints (e.g. poor drug distribution systems), concern among nurses about the quality of HIV care at some PHC sites, and lack of local area management support for and coordination of decentralisation.

The implementation of nurse-initiation was also influenced by a number of constraints. At some sites where nurses were re-prescribing and some HIV care had been successfully decentralised, a lack of clinical confidence and poor physician support appeared to be barriers to progressing to, and sustaining, full NIMART. In two sites, patient deaths and complications shortly after starting ART undermined nurse confidence and led to the temporary suspension of NIMART:

".’..when you see things happening [clinical complications] then you begin to feel somehow guilty to say, maybe, if you were not given this thing and they have been doing it the old way, maybe this couldn’t have happened. But on the other hand, when we see our patients doing well, then you feel proud.. at times, the clients come back to say, ‘You have brought my life back,’ and thank you for that. So those are some of the things that I have seen…There is the good and there is the other side.’ [STRETCH site manager and nurse]"

In other sites, a number of drug delivery issues and infrastructure deficits affected both initiation and maintenance of patients on ART. These are discussed below.

### Logistic and infrastructural constraints

In this section, we consider in more detail some of the above-mentioned logistical and infrastructural constraints. The STRETCH intervention took place against the background of a health system that was contending with a variety of difficulties, including resource constraints, pharmacy re-organisation, information system and transport problems, inadequate size of clinic buildings for the increasing numbers of patients, and, in some facilities, no functioning toilets or telephones. The new workload arising from providing NIMART further strained many of these key areas of logistics and infrastructure.

Nurses in all facilities said repeatedly that paperwork demands in the health system as a whole were onerous, and had been increased by NIMART. It is likely that the increase in administration work for NIMART was related to the increased numbers of ART patients rather than unusually intense recordkeeping for ART patients. This burden was compounded by weak and fragmented information systems that were insufficiently staffed and resourced.

STRETCH did reduce the need for referrals and patient transport between sites, an issue of urgent concern among most nurses and patients. One nurse at a PHC referral site described their difficulties referring patients to other distant sites for ART:

"‘…if the patient is very ill, then the transport is a problem. And the transport leaves early. Four o’clock in the morning…I had a case last week when one of the patients died there at [Clinic X] because she was very [too] ill to go. And then the complaint in the morning was that it was too cold to wait for the transport. So she died there. It was a new patient.’ [PHC referral site nurse]"

However, nurses reported that the decentralisation of monthly medication to local clinics, a key way to ensure fewer referrals and patient journeys, had been particularly stressful. This was a consequence of the national requirement that ART be dispensed under direct supervision of a pharmacist. This requirement was implemented by prepacking specific prescriptions centrally for named patients. These named prescriptions and packets of drugs then had to be transported back and forth between facilities. Poor communication and transport between pharmacy and clinic services made drug supplies unreliable. STRETCH also introduced significant drug storage and management challenges at smaller sites that already had insufficient space allocated to their pharmacy.

In other sites, unreliable delivery of ART drugs from hospitals and the central dispensing unit, as well as infrastructure deficits such as non-functioning telephone lines, were reported to have had significant effects on patients already on ART and made it very difficult for nurses to initiate new patients onto treatment. These were the obstacles cited by the two STRETCH intervention sites that had not progressed to phase three two years into the trial.

The Free State placed a temporary moratorium on ART initiation between November 2008 and February 2009 because of inadequate funding for ART procurement. This also impacted adversely on nurses’ morale. However, depletion of clinics’ drug stocks and other service disruptions are not unusual in this setting and STRETCH sites reported having dealt with the backlog of new patients needing treatment soon after the moratorium ended. Although the trial coordinator reported that some facilities struggled to return to nurse-initiation of ART, the moratorium was not reported as a factor leading to the slow progression of some sites through the three trial phases.

In general, though increased paperwork demands and weak IT systems were the subject of frequent criticism, these were not reported to have significantly affected implementation and were counter-balanced by improvements in other aspects of patient care such as fewer referrals and less transport requirements. Rather, a more important source of frustration and delay was pharmacy-related logistics and infrastructure challenges. These did not seem to significantly affect ongoing implementation of services in the medium-term but did contribute to initial delays and frustrations and the slow progression to full nurse-initiation in some sites.

### Human resources: increased workloads but short of spare capacity

The overall shortage of all categories of PHC workers was seen as a critical issue in every site. This included shortages of nurses and physicians as well as of pharmacists, managers, social workers, data clerks, lay counsellors, and administrative support. STRETCH increased nurse workloads through shifts to nurses of physician tasks, and also increased workloads on other team members through improvised shifting of duties as a result of this broader lack of capacity. For example, data capturers performed basic nursing duties (like weighing patients) when nurses were very busy or nurses dispensed when pharmacists were not available.

The initial enthusiasm for NIMART was tempered in some STRETCH sites by the increased workload, in particular from ongoing monthly patient follow-up visits. This workload curbed the drive to place new patients on treatment:

"‘We don’t want to promote it [HIV testing and ART] worldwide and then we can’t handle the load. So the promotion is basically through other people that were successful that’s come in…if we get somebody in the clinic identified as HIV-positive, we give them the information about ARVs. Personally, I think we must have a much bigger promotion for ARVs to make people aware that there is help…but as I say, we are not doing it because we can’t handle the burden if it’s much bigger.’ [local area manager]"

Nurses also reported that ART patients required more complex and comprehensive clinical input thereby increasing the time and effort required per consultation. This extra input was felt to be needed in particular at clinics that already had high patient volumes.

In contrast with increased nurse workloads, there were clear decreases in physicians’ routine workloads, suggesting that the underlying objective of NIMART was attained. For example, one physician was able to start seeing patients at other clinics because his work had been reduced significantly at the intervention site. The focus of physicians’ work also shifted, from managing all new cases and follow-up visits to managing only those cases with clinical complications:

"‘In the beginning … we started in 2006 as an ARV site here, and back then I obviously did virtually everything…But then, after the STRETCH started, it made my work much easier, especially the follow-ups because the sisters are now doing everything, and is just referring the problem cases to me. The workload is much less now … the paperwork, I would say.’ [physician at STRETCH site]"

Despite the growing and increasingly complex workload and the human resource constraints, nurses in the STRETCH sites described a substantial emotional reward from their investment in the NIMART programme and a long-term commitment to their patients (as noted in an earlier study in this setting [24]). This commitment to patients and optimism about treatment, however, was not limited to STRETCH sites. Even in control sites, where decentralisation of HIV services had started to improve treatment access, nurses reported the same kinds of emotional satisfaction:

"‘There’s hope now. In the past we could only give the Bactrim [an antibiotic] and say, ‘there’s no hope and that is it.’ Now at least we can say, ‘okay, if your CD4 is this and this, you can give this and that for it’…So it gives us a little option, and you can see the difference in patients. Some are being pushed in wheelchairs and are malnourished … you know, they are very sick, and then after three months the same patients walk in here and they start working again. You know, looking for work and planning their lives again…That’s making it ….worth it.’ [control site nurse]"

Nurses felt, however, that commitment often had negative effects for their own wellbeing. In particular, they felt that they needed much more support to sustain their clinic work, and that middle and upper management layers were uninterested, or unable to provide this support, or both.

### Challenges in supporting the development of clinical confidence among nurses

The STRETCH intervention included several forms of clinical support to ensure quality of care and to develop nurses’ clinical self-confidence. Physicians were supposed to provide support to nurses at clinic level by accepting telephone queries, providing feedback on referred cases, and visiting nurses on-site to discuss cases. Nurses were also expected to support and share skills with each other as HIV care shifted from a specialist to a generalist service.

It was also intended that STRETCH trainers would build clinical knowledge and confidence through ongoing training and support for nurses, both telephonic and in person. The local STRETCH support teams were supposed to complement this direct clinical support with assistance in addressing management and logistics challenges. The STRETCH trial coordinator was available to facilitate and encourage these relationships in this decentralised support system and to provide additional logistical and clinical advice where necessary. Further clinical support was available via a telephone hotline from the provincial ART programme’s ‘Centre of Excellence,’ though some nurses reported being too intimidated to call an expert whom they did not know and found it easier to contact the trial coordinator for advice.

Nurses were generally familiar and satisfied with the STRETCH approach to guidelines, given their prior experience with PALSA PLUS. However, they expressed concerns regarding the complexity of ART. While they agreed that HIV/AIDS should be treated like any other chronic disease, they also maintained it was more complex in terms of time per consultation, medication side-effects, and emotional involvement. There were some nurses who would therefore have preferred NIMART to have remained a vertical programme so that they could develop expertise within this specialty.

For the most part, though, initial resistance to STRETCH soon gave way to an acceptance of the viability and preferability of generalised nurse-initiation of ART. For some, provision of ART also brought with it a feeling of accomplishment and a degree of prestige among the other nurses and the patients for being able to offer this valuable service:

"‘You know, it’s an eye-opener, and it’s giving us an opportunity to be able even to use our brains even further. As I’ve said, you’ll be consulting and doing this. And there are times that you have to think deeper. And it’s even broadening our intelligence…to me, it’s an achievement, and it’s something that really boosted my confidence.’ [STRETCH nurse]"

Of greater concern for most nurses, however, were the volume of patients they had to see and their sense of being at the limit of their capacity to safely manage a rapid roll out. The STRETCH guidelines played an important role in this context, offering a secure platform for developing clinical confidence.

Two important forms of horizontal support within clinics emerged. Firstly, peer support among nurses appeared to be widespread and was effective in sites where no physicians were available. Secondly, nurses generally reported trusting and effective relationships with physicians in the sites where physicians were permanently employed or visited regularly. However, nurses at STRETCH sites that did not have a regular on-site physician presence generally ended up with little senior clinical mentorship.

Key reasons for inconsistent physician support included an insufficient number of physicians, poor coordination between physicians and STRETCH trainers, and a lack of relevant ART experience or interest in ART among physicians (particularly newly qualified physicians). Also some physicians worked only at distant treatment sites and did not visit the local assessment sites. In the early stages of implementation, friction between some physicians and nurses about treatment protocols resulted in insecurity among nurses, but the trial coordinator was able to mediate these conflicts.

Nurses generally felt that this gap in clinical support was not sufficiently addressed by the STRETCH trainers. They argued that STRETCH trainers were often not adequately experienced in ART ‘on the ground’ and were not able to provide the ongoing training and support that nurses needed. These trainers were generally drawn from a pool of middle managers with the expectation that they would supervise and support nurses after the initial training, rather than provide ongoing clinical training. Once nurses had developed some clinical confidence and experience, this supervisory aspect of their relationship with the STRETCH trainers was more effective. Again, the trial coordinator often addressed this gap, serving as an important source of clinical support both telephonically and in person.

Overall, however, it was the trial coordinator who had the most impact on ensuring clinical support and on developing the confidence of nurses at the intervention sites. Even though her efforts to address logistical and management challenges and provide direct clinical support went further than originally planned, her input was most intensive towards the beginning of the trial and tapered off as these issues were addressed. We discuss later the implications of her contribution for the broader sustainability of the STRETCH programme.

### The critical role of effective local and district management

Though STRETCH was well regarded by most managers throughout the health system, their input was at times insufficient to address the many day-to-day challenges of logistics, human resources, and clinical management, referral, and support. For example, during the course of the pre-intervention visits, STRETCH support teams were formed within each facility. These teams were composed of nurses, site managers, ART and pharmacy coordinators, and physicians. Management of these teams, however, was inconsistent and their success generally depended on the availability and commitment of the more senior staff members. Similarly, local area managers, whose input was often key to solving small-scale but crucial logistical problems, found it difficult to visit clinics under their jurisdiction, often because they had no budget for vehicles and fuel.

Managers at the facility level were expected to oversee general changes in service organisation and to assist with logistical arrangements, local intervention tailoring, and scheduling, guided by the STRETCH Implementation Toolkit. However, the trial coordinator reported that, on its own, the toolkit was often not enough to promote progress through the trial phases at sites that were not otherwise highly motivated. The coordinator found that she needed to work through many of the logistical and administrative issues directly with sites early in the implementation process.

The direct support by adequately informed and engaged managers at higher levels of the health system, for example at sub-district and district levels, and of district-based ART coordinators also facilitated the roll-out. In some sites, these managers were central to putting in place the elements that would allow for effective decentralisation. For example, decentralising routine HIV care within a district meant moving a number of tasks and responsibilities to new sites and staff. These included initial laboratory workup, drug readiness training, and the monthly supply of ARVs. The involvement of these managers, however, was highly variable, with some sites requiring much more contact with and support from the trial coordinator. Staff turnover within management was also a problem at all levels within the province.

Allowing sites to manage the pace of implementation eased management conflicts and improved problem solving. This bottom-up approach also appeared to increase ownership of the intervention by nurses and managers.

## Discussion

The accelerating roll out of ART in sub-Saharan Africa has reinforced the need to develop capacity for delivery at both the clinical and health systems levels, and to integrate comprehensive HIV/AIDS care into primary care. Our earlier work in this setting successfully addressed this by training all nurses in the clinical management of HIV/AIDS and respiratory diseases [13]. While this earlier training intervention improved adult HIV/AIDS and tuberculosis primary care, an accompanying process evaluation highlighted that nurse training alone could not address key health systems level constraints, including insufficient human resources and inadequate support for clinical care delivery and for planning and management [11]. The subsequent STRETCH intervention therefore combined: nurse training with task-shifting of diagnosis, treatment initiation, and most follow up care from physicians to nurses; the integration of HIV care into primary care services; the establishment of local management teams; and an implementation toolkit [17]. This study has examined the implementation of STRETCH to assess the acceptability of this approach and its interactions with health systems level factors.

### Building professionals’ acceptance of, and clinical self-confidence to deliver, NIMART

NIMART was generally well accepted by nurses. Its reception was less consistent among physicians, managers and other health service staff. Where resistance did emerge, it tended to be in sites where staff felt that they did not have adequate capacity to handle the management and logistical challenges of nurse-initiation, or which had health system problems that transcended HIV care. As in the earlier PALSA PLUS study [11], their concerns about implementing NIMART were, therefore, more about health system constraints than about clinical practice. Similar findings have been reported in other programmes in this setting and elsewhere [1,25-28].

The study identified a number of factors that influenced nurses’ clinical confidence to implement NIMART. These included: the use of nurse-specific guidelines that included clear referral protocols; familiarity with the guideline-based approach to care; phased implementation of the intervention with the pace set by the individual clinics; and effective training and clinical support and supervision from physicians either on-site or off-site. The data suggest that a key weakness in developing the clinical confidence of nurses was inadequate clinical support. As we discuss in more detail below, this was related more strongly to systems-level factors, such as the large number of other responsibilities of trainers and logistic difficulties in travelling to clinics, rather than to an unwillingness or inability on the part of physicians or trainers to provide the input needed. Furthermore, there were few experienced ART supervisors and physicians who could be recruited to support nurses at the start of the programme.

Our findings also indicate the importance of striking a balance between the recommended implementation stages and clinical pathways set out clearly in the STRETCH toolkit and the guidelines, respectively, and allowing flexibility in implementation and timing at individual sites. This flexibility resulted in substantial variation across sites in the pace of implementation. However, it also allowed for the implementation of NIMART to combine fidelity of the overall approach with effective adaptation to local needs and contexts. A comparison of ART scale-up in three South African provinces has also highlighted the importance of a balance between fidelity to the core elements of a programme and freedom to tailor delivery approaches to the local context. Writing about ART provisions in the same Free State context three years earlier, Schneider et al. argue that a rigid and cautious approach to ART implementation kept coverage low [28].

### Task-shifting requires broader organisational transformation

At the organisational level, STRETCH required much more than the shifting of ART initiation and management tasks from physicians to nurses. It also involved changes in many other roles, relationships and responsibilities both within clinics and in the health system more widely. For example, nurse-initiation also required strengthened clinical relationships between nurses and physicians so as to facilitate the referral by nurses of difficult ART cases to physicians. Similarly, task-shifting and the integration of ART services into PHC entailed corresponding shifts in workflows and duties for other clinic-level staff and therefore required increased communication and coordination among a wide range of healthcare providers and managers. Where this communication was weak, strains emerged in the working relationships between physicians and nurses and between the other staff involved in ART and HIV care such as pharmacists, data entry clerks and lay counsellors. These complex consequences of task-shifting at the organisational level were not fully anticipated in the design of the STRETCH intervention.

A key factor in successfully addressing these challenges was the presence of the STRETCH trial coordinator during the initial stages of the implementation. The trial coordinator acted as a ‘problem-solver’ and negotiated issues across the health system. As we have noted elsewhere, the STRETCH coordinator also functioned as an ‘agent of change’ in this intervention, playing a role in facilitating the active participation of staff in implementation. These roles have been acknowledged as important functions of external facilitation of the implementation of complex health interventions [17,29]. The support offered by the trial coordinator was much wider than originally anticipated and highlighted the many system-level gaps and points of friction that were potentially critical barriers for implementing NIMART.

This evaluation has several limitations. First, its findings may need to be generalised with caution to other settings which differ in their health systems organisation and capacity and in their professional norms and practices. Many of the key factors identified here, however, as having an impact on task-shifting for scale-up of NIMART will be similar in other resource-constrained primary care settings. Second, observation of clinical and management/supervisory practices were limited and the findings therefore rely more on stakeholders’ views regarding the implementation of STRETCH. The range of stakeholders consulted, however, allowed for triangulation and strengthening of the analysis.

Many of the constraints identified are at a systems level and are likely to be relevant to other instances where complex health systems interventions are implemented. Similarly, many of the enablers of this intervention—for example treatment protocols, effective support, and phased implementation—might be broadly applicable to other interventions. It may be, however, that the socio-political meanings and emotional urgency attached to NIMART benefited this programme and it is not clear whether other health programmes would enjoy the same potential advantage.

## Conclusions: lessons for the scale-up of NIMART elsewhere in South Africa and in other HIV/AIDS high-burden settings

A number of potentially widely applicable insights emerged from this study regarding the scaling-up and sustainability of interventions like STRETCH. Most importantly, while nurse-initiation of ART appears to be a largely acceptable form of task-shifting and did not require nurses to work in ways that were dramatically different from other aspects of their clinical practice, it also represents a significant re-organisation of health services. In contexts that combine high HIV/AIDS prevalence and weak health systems, scaling-up and sustaining such service re-organisation is a substantial undertaking whose success depends on a number of factors being in place at different levels of the health system as well as attention to the reasons underlying specific forms of professional practice [30].

How to embed or normalise NIMART in primary care practice therefore needs careful consideration in each context [31]. In our study, for example, the complex legislative and policy environment for the distributions of ART represented a significant barrier to scaling up. This policy required that ARVs be dispensed by or under direct supervision of a pharmacist. As most PHC facilities did not have pharmacists, ARV prescriptions had to be dispensed centrally into patient-named packets and transported every month to the PHC clinic to be issued to the patient.

Because implementation of NIMART necessitated a range of health system changes, a too rigid or rapid full-scale implementation of NIMART would risk neglecting some of the key elements of the STRETCH intervention that facilitated adjustments at the clinic and health systems levels. For example, in contrast to recent national policy changes requiring nurses across South Africa to initiate ART within a set timeframe, STRETCH allowed nurses and managers to develop local solutions to logistical problems, take time to build their confidence, and progress through the different stages of STRETCH at their own pace.

Other important elements of STRETCH included clear clinical guidelines and referral protocols, the availability of several sources of clinical support for nurses new to ART, and the presence of strong local management support and problem solving [7,32]. Several recent studies have similarly highlighted the important potential benefits of protocol-based care [33] and proper clinical supervision in the primary healthcare context [34] to increase the scope and autonomy of nurses’ work. However, having a physician or experienced nurse available to provide clinical support is not necessarily a simple change and involves further task-shifting of roles upwards that needs to be anticipated by programme designers. Researchers have also noted challenges in using clinical protocols [33] as well as the relative lack of evidence for how protocols can be effectively implemented in practice [35].

The good news is that most of these problems with scale-up lessened over time. As this intervention has matured in the STRETCH sites and nurse-initiation has become normalised into the health system, the need for external management or clinical support has decreased. Support from the trial coordinator in particular tapered off as clinical confidence grew, systems difficulties were addressed, and NIMART was integrated into routine practice. These findings suggest that NIMART is feasible and acceptable, and this in turn suggests that the approach may also be useful in other sub-Saharan African contexts where primary care is delivered primarily by nurses. However, this intervention is complex to implement, requires significant reorganisation of health services and ongoing support, and does not in itself solve broader health systems challenges.

## Abbreviations

ART, Antiretroviral treatment; ARV, Antiretroviral; LMIC, Low and middle income countries; PHC, Primary healthcare; NIMART, Nurse-initiated and managed ART; PALSA PLUS, Practical approach to lung health and HIV/AIDS (South Africa); STRETCH, Streamlining tasks and roles to expand treatment and care of HIV.

## Competing interests

The Knowledge Translation Unit of the University of Cape Town Lung Institute (DG, LF, BD, and EB) provides training in PALSA PLUS/STRETCH to the South African and Western Cape Departments of Health. The authors declare no other competing interests.

## Author contributions

SL, CJC, DG, KU, LF, MB and MZ participated in the design of the study. DG, CJC and SL collected and analysed the data, interpreted the results and drafted the manuscript. KU, MB, BD, LF, MZ and EB contributed to revisions of the manuscript. All authors read and approved the final manuscript.
